# Prospective Study of BK Virus Infection in Patients with Inflammatory Bowel Disease

**DOI:** 10.1155/2014/970528

**Published:** 2014-02-13

**Authors:** Virginia Flores, Belén Rodríguez-Sánchez, Ignacio Marín-Jiménez, Emilio Bouza, Luis Menchén, Patricia Muñoz

**Affiliations:** ^1^Department of Digestive Diseases, General University Hospital Gregorio Marañón, Dr. Esquerdo 46, 28007 Madrid, Spain; ^2^CIBER Hepatic and Digestive Diseases (CIBEREHD), 07110 Bunyola, Balearic Islands, Spain; ^3^Department of Clinical Microbiology and Infectious Diseases, General University Hospital Gregorio Marañón, Dr. Esquerdo 46, 28007 Madrid, Spain; ^4^CIBER Respiratory Diseases (CIBERES CB06/06/0058), 07110 Bunyola, Balearic Islands, Spain; ^5^Department of Medicine, Faculty of Medicine, Complutense University of Madrid, 28040 Madrid, Spain; ^6^Spanish Study Group on Infection in Transplant Recipients (GESITRA-RESITRA-REIPI), RD06/0008/1025, Spain

## Abstract

Patients with inflammatory bowel disease (IBD) have an immune-deficient baseline status further modulated by immunosuppressive therapy that may promote the reactivation of latent viruses such as BK virus (BKV). The aim of this prospective study was to determine the prevalence of BKV infection in IBD patients and its potential relationship with the immunosuppressive treatment. Paired urine and plasma samples from 53 consecutive patients with IBD and 53 controls were analyzed. BKV detection was performed by conventional PCR and positive samples were further quantified by real-time PCR. No viremia was detected. BKV viruria was significantly more common in IBD patients than among the controls (54.7% versus 11.3%; *P* < 0.0001). The only risk factor for BKV viruria in IBD was age (47.2 ± 16.3 versus 37.8 ± 15.2; *P* = 0.036), and there was a trend towards higher rate of viruria in outpatients (61.5% versus 38.5%; *P* = 0.096) and in those not receiving ciprofloxacin (59.5% versus 40.5%; *P* = 0.17). A clear impact of the immunosuppressive regimen on BKV infection could not be demonstrated.

## 1. Introduction

Human polyomavirus BK (BKV) is a double-stranded DNA virus with a small circular genome. It belongs to the Polyomaviridae family, which includes JC virus, Simian Virus 40 (SV40), and Merkel cell polyomavirus [1, 2]. These viruses are very similar in structure, with high DNA sequence homology and an icosahedral capsid.

More than 80% of the world's population has serological evidence of exposure to BKV [3]. Primary infection typically takes place during childhood, with no specific symptoms, and the virus remains latent for life, mainly in urothelial cells [4]. The natural route of transmission has not been clearly defined and may be oral or respiratory. The virus has been isolated in urine, plasma, and biopsy specimens such as kidney, liver, eye, and brain [5]. Under immunosuppressive conditions, BKV can reactivate and cause disease. This is common in kidney recipients and AIDS patients, in whom BKV may cause renal failure due to severe acute interstitial nephritis, meningoencephalitis, pneumonitis, and retinitis [5, 6]. In hematopoietic stem cell recipients the most common manifestation of BKV is hemorrhagic cystitis [7]. Besides these direct effects, an oncogenic potential has been reported in patients with colorectal cancer [8]. Regarding gastrointestinal manifestations, a case of colonic ulceration associated with BKV has been published [9]. Its role in other immunocompromised populations has not been elucidated.

In this study, we analyze the prevalence of BKV infection in patients with inflammatory bowel disease (IBD), a condition that has not previously been related to BKV. We also analyze the potential relationship between BKV infection and the different immunosuppressive regimens used in this population.

## 2. Material and Methods

### 2.1. Patients

We performed a prospective study in 53 consecutive unselected IBD patients (inpatients and outpatients) who attended consecutively in the Digestive Medicine Department between April 2008 and May 2012. Patients were admitted to hospital for exacerbation of their disease or attended in the day-hospital for clinical follow-up. Plasma and urine samples from 53 healthy volunteers, matched by age and gender, were also included as a control group.

Using a preestablished form, the same trained physicians collected epidemiological and clinical data from all patients as follows: IBD activity and extension, clinical characteristics, family and personal history, age at diagnosis, perianal disease, need for surgery, renal insufficiency, extraintestinal disease, and previous and current therapy. Ciprofloxacin was administered to patients with acute outbreaks due to exacerbation of their disease. This antibiotic was chosen for its anti-inflammatory effect on the intestinal mucosa and to prevent bacterial overinfections.

The severity of the underlying disease was classified according to standard definitions, including the Harvey-Bradshaw index and the Montreal classification for inflammatory bowel diseases [10, 11]. For analytical purposes, we considered as “severe” the forms classified as such with the Harvey Bradshaw index for Crohn's disease and as S3 for those with ulcerative colitis.

Renal function was assessed using plasma creatinine levels and glomerular filtration rate (GFR) determined using the MDRD equation. Renal impairment was defined as the increase in the serum creatinine × 2. Microscopic hematuria was defined as the presence of ≥3 RBCs per high-power field in at least 2 specimens of unspun urine. Pyuria was described as >3 WBC/hpf unspun urine or positive leukocyte esterase.

BKV infection was defined as the evidence of virus exposure reflected by the PCR detection of virus in blood or urine samples. BKV disease was defined as the histopathological or ultrastructural evidence of cytopathic and organic damage caused by this virus [3].

The local ethics committee approved the study and all patients gave their informed consent.

### 2.2. Nested and Quantitative Real-Time Polymerase Chain Reaction (PCR)

Simultaneous urine and plasma samples were collected for detection of BKV. All samples were aliquoted and frozen at −20°C prior to analysis. DNA was extracted using the QIAamp viral DNA mini kit (Qiagen, Hilden, Germany) and stored frozen at −70°C before use. DNA samples were screened for BKV using a nested PCR assay, which amplified a specific region of the BKV large T-antigen [12]. The methodology of this PCR assay is addressed in detail elsewhere [13–15].

Positive samples were quantified using LightCycler chemistry (Roche Diagnostics, Mannheim, Germany) according to a previously described real-time assay based on a region of BKV VP1 (detection limit, 100 copies/mL) [16]. Known concentrations of the plasmid pB-VP1 containing the amplified BKV region (kindly provided by Professor Kwong from the University Hospital of Hong Kong) were used to obtain a standard curve for quantification.

### 2.3. Statistical Analysis

The characteristics of patients with and without BKV infection were analyzed. Normally distributed quantitative variables were compared using a *t*-test and mean results with standard deviation were provided; nonnormally distributed variables were compared using the Mann-Whitney *U* test. Normal distribution was analyzed with the Kolmogorov-Smirnov test. Qualitative variables were compared using the Fisher exact test. All *P* values were based on a 2-tailed test of significance (*P* < 0.05). Statistical analysis was performed with SPSS version 15.0.

## 3. Results

### 3.1. Study Population

The study sample comprised 53 patients with IBD. The underlying conditions consisted of Crohn's disease (32, 60.4%) and ulcerative colitis (21, 39.6%). The mean age of the patients was 42.92 ± 16.34 years (median 33 years, 15–72), and 49.1% were men.

The classification of the severity of the underlying diseases is shown in Table 1. Forty-six patients had moderate disease (86.8%) according to the Harvey-Bradshaw index and the Montreal classifications [10, 11]. Fourteen patients were on an acute exacerbation episode and were hospitalized, and the remaining thirty-nine were outpatients on chronic follow-up (Table 2).

Regarding immunosuppressive therapy, 12 (22.6%) patients were not receiving any immunosuppressive agent since they were in remission. The remaining 41 (77.4%) received infliximab/adalimumab, 24 patients; azathioprine, 9 patients; and systemic corticosteroids, 8 patients.

Renal function was normal (mean creatinine, 0.80 ± 0.18 mg/dL; median 0.8, 0.5–1.4) in all but 1 patient who fulfilled our criteria for renal insufficiency. This patient had left-sided nonsevere ulcerative colitis (treated with azathioprine) and presented BK viruria, although with a low viral load (100 copies/mL). No relationship between BKV infection and renal function parameters was found in this population.

### 3.2. BKV Prevalence and Viral Load

None of the IBD patients developed viremia. The prevalence of BKV viruria was significantly higher in the study group than in the control group (29 IBD patients-54.7%-versus 6 healthy volunteers-11.3%-; *P* < 0.0001).

When IBD patients with and without viruria were compared, higher rates were observed in older patients (47.2 ± 16.3 years versus 37.8 ± 15.2 in nonviruric;  *P* = 0.036). A trend towards a more frequent BKV viruria was found in patients not receiving ciprofloxacin (59.5% versus 40.5%, *P* = 0.17) and in outpatients (61.5% versus 38.5%; *P* = 0.096). Outpatients received less frequently systemic corticosteroids (16.7% versus 57.1%; *P* = 0.095).

We could not find a relationship between BKV viruria and the underlying disease (Crohn's disease versus ulcerative colitis; *P* = 0.610), the severity of the IBD disease (*P* = 0.890), previous surgery (*P* = 0.930), or treatment with an immunosuppressive drug (*P* = 0.302) (Table 2).

The median viral load of viruric patients was 2log10 genome equivalents (GEq)/mL (25th percentiles = 2log10 GEq/mL, 75th percentile = 5log10 GEq/mL). A high viral load, defined as 3.5 GEq/mL [17], was detected in 5 patients (median 5, 2log10 GEq/mL). High viral load was more frequent in younger patients (mean age 37.6 ± 11.8 years versus 48.4 ± 17.5 years; *P* = 0.203) and in patients receiving monoclonal therapy (28.6% versus 6.7%; *P* = 0.119) (Table 3).

## 4. Discussion

This is the first study on BKV infection in patients with IBD. We observed that 54.7% of IBD patients were viruric, a significantly higher frequency than a control population, although a relationship with attributable clinical manifestations could not be established.

The frequency of BKV viruria in IBD is higher than the rate expected for the age range of the study population [17]. It could be explained by the baseline immunosuppressive status of patients with IBD and by the use of immunosuppressive drugs necessary for the treatment of the illness. 41.7% of viruric patients were not receiving any immunosuppressive agent, while 58.3% were receiving anti-TNF treatment. In addition, BKV viral load tended to be higher in patients receiving infliximab/adalimumab (5log10 GEq/mL versus 3, 5log10 GEq, *P* = 0.119), although no statistical significance was reached probably due to the limited number of patients with high viral load.

Some monoclonal antibodies have been demonstrated to reactivate polyomaviruses. Severe forms of progressive multifocal leukoencephalopathy due to JCV, another *Polyomavirus, *have been described in multiple sclerosis patients treated with the humanized monoclonal anti-alpha-4 integrin antibody, natalizumab, and 5 cases of BKV CSF infection [18, 19]. Asymptomatic reactivation of BKV has also been described in patients with multiple sclerosis treated with natalizumab and the frequency of BKV viruria increased with the number of doses of natalizumab received (mean, 11.2 doses) [20]. However, we could not demonstrate a similar relationship of infliximab/adalimumab with BKV, and none of our patients developed attributable neurological manifestations.

In our series, BKV infection was not associated with the severity of IBD. In fact, BKV infection was more prevalent in outpatients, who had less severe disease and were receiving less frequently corticosteroids (16.7% versus 57.1%, *P* = 0.095) and ciprofloxacin (59.5% versus 40.5%, *P* = 0.17). Ciprofloxacin has been associated with lower rates of BK viremia in renal transplant and hematopoietic transplant recipients [21–23]. This is due to its capacity to inhibit the polyomavirus large T-antigen helicase [24]. In our study, IBD patients that were receiving ciprofloxacin were less prone to have an infection caused by BKV (36.4% versus 63.6%; *P* = 0.17).

Finally, there was not any relationship between renal dysfunction and BKV viruria in this population. Only 1 patient had renal insufficiency, although it was present before BKV infection, and neither this patient nor the rest of the population developed further renal impairment. However, other types of immunocompromised populations, such as renal transplant recipients or AIDS patients, develop an increased rate of interstitial nephritis and renal failure associated with BKV infection [6].

Our study has some limitations. The relatively low number of patients prevented us from establishing a statistical relationship between the use of monoclonal antibodies and BKV reactivation and finding related clinical manifestations.

## 5. Conclusion

We observed that IBD patients have a high rate of BKV viruria and that it is more frequent in outpatients and in those not receiving ciprofloxacin. More studies are needed to determine the role of BKV in this population.

## Figures and Tables

**Table:** (a) Patients with Crohn's disease (*n* = 32)

Age at diagnosis	Location	Behavior	Perianal disease	Harvey bradshaw
A1: 3 (9.4%)	L1: 8 (25.0%)	B1: 13 (40.6%)	No: 21 (65.6%)	Remission: 8 (25.0%)
A2: 24 (75.0%)	L2: 8 (25.0%)	B2: 8 (25.0%)	Yes: 11 (34.4%)	Mild: 9 (28.1%)
A3: 5 (15.6%)	L3: 15 (46.8%)	B3: 11 (34.4%)		Moderate: 10 (31.2%)
	L4: 1 (3.2%)			Severe: 5 (15.7%)

**Table:** (b) Patients with ulcerative colitis (*n* = 21)

Extension	Severity
E1: 5 (23.8%)	S0: 5 (23.8%)
E2: 7 (33.3%)	S1: 6 (28.6%)
E3: 9 (42.9%)	S2: 8 (38.1%)
	S3: 2 (9.5%)

A1: below 16 y; A2: 17–40 y; A3: above 40 y.

L1: ileal; L2: colonic; L3: ileocolonic; L4: isolated upper disease.

B1: nonstricturing, nonpenetrating; B2: stricturing; B3: penetrating.

E1: ulcerative proctitis. Involvement limited to the rectum.

E2: left-sided ulcerative colitis. Involvement limited to a proportion of the colorectum distal to the splenic flexure.

E3: extensive pancolitis. Involvement extends proximally to the splenic flexure.

S1: mild colitis; S2: moderate colitis; S3: severe colitis.

**Table 2:** Comparison of inflammatory bowel disease patients with and without BKV viruria.

	All patients	Viruric patients	Nonviruric patients	*P* value
	*N* = 53	*N* = 29	*N* = 24	
Age (mean ± SD)	42.92 ± 16.34	47.2 ± 16.3	37.8 ± 15.2	**0.036**
Males (%)	26 (49.1)	12 (46.2)	14 (53.8)	0.217
Type of IBD				
Crohn's disease (%)	32 (60.4)	17 (53.1)	15 (46.9)	0.610
Ulcerative colitis (%)	21 (39.6)	12 (57.1)	9 (42.9)	
Previous surgery (%)	18 (34.0)	8 (44.4)	10 (55.6)	0.930
Severity of IBD				
Severe	7 (13.2)	4 (57.1)	3 (42.9)	0.890
Nonsevere	46 (86.8)	25 (54.3)	21 (45.7)	
Hospital admission at inclusion (%)				
No	39 (73.6)	24 (61.5)	15 (38.5)	0.096
Yes	14 (26.4)	5 (35.7)	9 (64.3)	
Immunosuppressive therapy (%)				
No	12 (22.6)	5 (41.7)	7 (58.3)	0.302
Yes	41 (77.4)	24 (58.5)	17 (41.5)	
Infliximab/adalimumab				
No	29 (54.7)	15 (51.7)	14 (48.3)	0.630
Yes	24 (45.3)	14 (58.3)	10 (41.7)	
Systemic corticosteroids				
No	45 (84.9)	23 (51.1)	22 (48.9)	0.771
Yes	8 (15.1)	6 (75)	2 (25)	
Serum creatinine (mg/dL, mean ± SD)	0.80 ± 0.18	0.82 ± 0.14	0.78 ± 0.2	0.411
Ciprofloxacin at inclusion				
No	42 (79.2)	25 (59.5)	17 (40.5)	0.170
Yes	11 (20.8)	4 (36.4)	7 (63.6)	
Urinalysis				
Hematuria	6 (11.3)	4 (13.8)	2 (8.3)	0.532
Leukocyturia	8 (15.1)	5 (17.2)	3 (12.5)	0.631

Statistically significant *P* values are shown in bold.

**Table 3:** BKV viral loads in the 29 BKV viruric patients with inflammatory bowel disease.

	Low viral load	High viral load	*P* value
	<3.5 log⁡GEq/mL	>3.5 log⁡GEq/mL	
Mean age	48.4 ± 17.5	37.6 ± 11.8	0.203
Crohn's disease			
Yes 17 (58.6)	14 (82.4)	3 (17.6)	0.945
No 12 (41.4)	10 (83.3)	2 (16.7)	
Moderate-severe IBD disease			
Yes 4 (13.8)	4 (100)	0 (0)	0.326
No 25 (86.2)	20 (80)	5 (20)	
Hospital admission at inclusion			
Yes 5 (17.2)	4 (80)	1 (20)	0.858
No 24 (82.8)	20 (83.3)	4 (16.7)	
Infliximab/adalimumab			
Yes 14 (48.3)	10 (71.4)	4 (28.6)	0.119
No 15 (51.7)	14 (93.3)	1 (6.7)	
Systemic or local corticosteroids			
Yes 8 (27.6)	7 (87.5)	1 (12.5)	0.677
No 21 (72.4)	17 (81.0)	4 (19.0)	
Use of quinolones			
Yes 4 (13.8)	3 (75)	1 (25)	0.658
No 25 (86.2)	21 (84)	4 (16)	
